# No short-cut in assessing trial quality: a case study

**DOI:** 10.1186/1745-6215-10-1

**Published:** 2009-01-07

**Authors:** Karim F Hirji

**Affiliations:** 1 Department of Epidemiology and Biostatistics, Muhimbili University of Health and Allied Sciences, P. O. Box 65015, Dar es Salaam, Tanzania

## Abstract

**Background:**

Assessing the quality of included trials is a central part of a systematic review. Many check-list type of instruments for doing this exist. Using a trial of antibiotic treatment for acute otitis media, Burke et al., *BMJ*, 1991, as the case study, this paper illustrates some limitations of the check-list approach to trial quality assessment.

**Results:**

The general verdict from the check list type evaluations in nine relevant systematic reviews was that Burke et al. (1991) is a good quality trial. All relevant meta-analyses extensively used its data to formulate therapeutic evidence. My comprehensive evaluation, on the other hand, brought to the surface a series of serious problems in the design, conduct, analysis and report of this trial that were missed by the earlier evaluations.

**Conclusion:**

A check-list or instrument based approach, if used as a short-cut, may at times rate deeply flawed trials as good quality trials. Check lists are crucial but they need to be augmented with an in-depth review, and where possible, a scrutiny of the protocol, trial records, and original data. The extent and severity of the problems I uncovered for this particular trial warrant an independent audit before it is included in a systematic review.

## Background

Clinical trials of poor quality continue to be reported in virtually all medical fields [1-6]. Assessing the quality of a trial is thereby essential, both for judging the reliability of its conclusions, and for including it into a systematic review. Due effort has, accordingly, been spent on developing appropriate instruments for the task. A 1999 overview found 25 different check lists for evaluating the quality of a clinical trial [7-10].

Several concerns about trial quality check lists have, however, emerged. One, they vary appreciably in terms of the number and types of components. Two, the weight they accord to the components differs markedly. And three, some lists have items unrelated to assessing bias, or generalizability of trial findings. The earlier use of quality scores as weights in a meta-analysis, or for quality ranking of trials is no longer advised. Recent work has sought to identify the components of quality that often or strongly affect trial outcomes. The findings show that adequate allocation concealment is a key component for all types of trials while the importance of the extent of blinding, even when feasible, varies from one medical field to another [10-17].

Systematic reviews now tend to use quality instruments with a few key components that have been well studied. The evaluation scheme noted in [18], page 58, which includes four items (method of treatment assignment, control of bias after assignment, blinding and outcome assessment), is one example. And, even with a good scheme, the evaluation ought to be performed by two or more independent, and when possible, blinded reviewers. Any discord is resolved by discussion.

Despite these positive strides, quality assessment of trials is still a nascent discipline. The sheer number of unevaluated published and unpublished trials, the problem of protocol deviation, the lack of standardized outcomes, discrepancies between reported and actual conduct of trials, and distortions induced by conflict of interest of necessity render its current conclusions tentative. The formulation of medical area specific quality instruments, and including quality components relating to external validity, furthermore, have yet to receive due attention [16,19-26].

There is also a methodologic matter that seems to have escaped notice. In the process of developing instruments with essential items, we may become oblivious to the fact that this approach as such discretizes a complex construct. It may hence foster an automated type of evaluation whereby for each trial, a quality review consists of an examination of the methods section, and a quick read of the rest of the paper with the focus on noting the presence or absence of relevant key words. There is then a danger that, even with independent and blinded reviewers using well regarded components of quality, such an approach may at times generate a highly misleading assessment of the quality of a clinical trial.

This papers aims to demonstrate, with the help of a case study, that the danger is real, not just theoretical. The case study pertains to the routine prescription of antibiotics for acute otitis media (AOM) in children. This has been and remains a common clinical practice. Yet, it has spawned extensive controversy in the medical literature. The fact that the clinical trials in that field had different selection criteria, rules of diagnosis, and outcome measures also contributed to the discord [27]. The quality of especially the early trials is a key concern [28]. The trial of van Buchem and colleagues [29], the related correspondence [30,31], two semi-supportive editorials [32,33], and a detailed critique [34], well illustrate the type and extent of the discord that has long prevailed for treating this common pediatric ailment.

The first comprehensive quality review, based on 24 methodologic criteria, of antibiotic related trials for AOM was published in 1992. Covering a total of 50 trials, it concluded: "*Many trials are methodologically flawed which makes it diffcult to accept their result. In view of current controversy on management of acute otitis media, well conducted placebo-controlled trials are still needed*." [35]. This assessment predates any systematic review for antibiotic treatment of acute otitis media.

The specific case I study is the clinical trial of amoxycillin for mild AOM in children, [36], published in 1991. Referred to from now on as Burke et al., it was a double-blind trial in which 114 children diagnosed with AOM were randomized to amoxycillin, and 118 to a placebo. Its message was unequivocal: "*Use of antibiotic improves short term outcome substantially and therefore continues to be an appropriate management policy*."

This trial was not covered in the comprehensive quality review of acute otitis media trials in [35]. But, since its publication, it has featured prominently in the systematic reviews for AOM, where it has also been subjected to quality evaluation. In this paper, I compile these evaluations from the nine relevant systematic reviews I identified. Further, I show, through an in-depth but unstructured dissection of Burke et al., that they all are highly misleading evaluations.

## Results and discussion

### Trial description

Burke et al. evaluated the efficacy of amoxycillin for AOM in children between the ages of 3 to 13 years. Unlike most previous AOM trials, this was a multi-general practice based study. To attain uniformity in diagnosis, evaluation and follow up, training sessions for the participants were held. Children were evaluated at the clinic (initially, day 8, one month, three months) and through two home visits during the first week. Long term (one year) evaluation was also done. Outcomes based on parental diaries, home visits and in-clinic assessments were analyzed.

The main features of the trial, extracted mainly from the Patients and Methods section of the paper, appear in Table 1. The inclusion and exclusion criteria were specified, and a form for implementing them was devised. The trial was sized for 80% power to detect a general effect. A double blind, placebo-controlled design with an adequately concealed randomization code was employed. Data analysis methods were declared, and the main analysis was said to follow the intent-to-treat principle. The rates of follow up in all the stages of the trial seem acceptable. Hence, other than having many outcome variables, it appeared to have had, in the main, the features of a good quality trial. This was the verdict I arrived at through a cursory check-list evaluation of Burke et al.

**Table 1:** A check-list based quality assessment of Burke et al

Feature	Assessment	Feature	Assessment
1. Study Question & Population	Defined	12. Patient, Care Giver & Assessor	Blinded
2. Treatment & Placebo	Described	13. Informed Consent	Obtained
3. Inclusion Criteria	Specified	14. Statistical Method	Specified
4. Exclusion Criteria	Specified	15. Statistician Author*	No
5. Main Outcome Measures	Listed, Many	16. Intent to Treat Analysis	Performed
6. Multipractice Study	Yes	17. Baseline Characteristics	Reported
7. Sample Size & Power	Calculated	18. Groups Similar at Baseline	Mostly
8. Missing Data & Losses	Allowed For	19. Dropouts Reported	Yes
9. Randomization Method	Described	20. Dropout Rate	Below 10%
10. Central Randomization	Yes	21. Reasons for Dropouts	Given
11. Randomization Concealment	Adequate	22. Findings Support Conclusion 3	Apparently

Note: Assessment done by the author with criteria from [19]; * Statistician acknowledged.

### Evaluation in systematic reviews

The nine systematic reviews used different schemes to assess trial quality. Two reviews used general quality criteria like randomization for trial selection but did not mention a further quality evaluation [37,38]. The seven reviews with a formal quality evaluation used instruments with three to eleven items. Three reviews gave a numeric quality score, of which, one just reported the distribution of scores [39-41]. Three reviews had independent evaluators, and in one, they were also blinded [39,41,42]. One review was an individual patient data meta-analysis with full access to trial data, including that of Burke et al. The data for all the included trials in this review were "*thoroughly checked for consistency, plausibility, integrity of randomization, and follow-up*" [43].

Table 2 summarizes the quality evaluations of Burke et al. in these reviews. (The details are in Additional file 1.) In relation to internal validity, only three reviews had one or two concerns for the trial [38,41,42]. These were: a possible lack of comparability of groups at baseline (crying), an unclear outcome variable, and unspecified problems with the follow up. These issues were noted in isolation, and not pursued in any detail. One highly critical review noted only one issue, and that in a superficial and biased way [38]. Overall, it was deemed a good quality trial. The three most recent systematic reviews including the individual patient data meta-analysis, in particular, declared it a high quality trial.

**Table 2:** Quality assessments of Burke et al. in systematic reviews

Review	Year	NCT*	Quality	Internal Validity Problems
1. Lehnert [37]	1993	5	Good	None Stated
2. Rosenfeld et al. [39]	1994	4	High	None Stated
3. Del Mar et al. [40]	1997	8	10/11	None Stated
4. Froom et al. [53]	1997	7	Adequate	None Stated
5. Cantekin [38]	1998	8	Not Good	Baseline Comparability
6. Marcy et al. [41]	2001	6	4/5	Dropouts Description
7. Glasziou et al. [42]	2003	10	High	Baseline Comparability, MO†
8. Rosenfeld [46]	2003	9	High	None Stated
9. Rovers et al. [43]	2006	6	High	None Stated

*NCT = Number of Controlled Trials; †MO = Misidentified Outcome

### Student evaluation

The results of the comparative two-trial evaluation by the students are in Table 3. Burke et al. was rated as a good or excellent study by nearly 90% of the student respondents while not a single student gave such a rating to the paper [44]. In comparative terms, these evaluations were consistent with those obtained from the systematic review [40].

**Table 3:** Student assessment: Burke et al. versus Halsted et al.

	Quality Score					
Study	Very Bad	Poor	Acceptable	Good	Excellent	Total
Halsted et al. [44]	3	4	4	0	0	11
Burke et al.	0	0	1	5	3	9

Note: 2 students had not read Burke et al.

### In-depth dissection

My comprehensive dissection of the paper, in contrast, found an extensive number of design pitfalls, protocol deviations, implementation glitches, data irregularities, analytic flaws and reporting biases that were overlooked or only partly noted in the three types of evaluations above, including the three most recent systematic reviews. These problems were serious enough to call into question all the main findings of the trial. Implementation related deficiencies affected baseline comparability, level of follow up, extent of missing data, and other key facets of the trial in an interrelated manner and introduced serious biases in the emergent data.

The exact sources and details of my dissection of Burke et al. are in Additional file 2. Each assertion made below is backed by requisite referencing, reasoning and/or computation given there. The reader is strongly advised to study it with due care to judge the adequacy of evidence provided. Now, I summarize my major findings. The tables cited in this subsection refer to the respective tables in Burke et al.

#### Trial design

As described above, Burke et al. generally had a good design. The three problems I found were: (i) the number of main outcome variables, twenty, was excessive, (ii) the designation of the set of main variables varied in different parts of the paper; and (iii) the computation of sample size had used abstract numbers, was in error by a margin of about 20%, and did not allow for missing data or multiple outcomes.

#### Trial conduct

Evidence for major deviations from protocol at the baseline visit, two home visits, and in-clinic visit on day 8 is persuasive. These deviations produced serious biases in the trial data. The medium and long term evaluations, on the other hand, seem to have fared according to plan.

(i) At baseline, at least 12% of the cases were recruited in violation of a key ethical and scientific exclusion criterion (bulging ear drums) set for the trial; 16 of the 17 study practices demonstrated poor record keeping; baseline data were not collected fully, and in particular, no data on fever at presentation-a key prognostic factor-were reported. Besides low level of completeness, the baseline data also had biased patterns. Thus, the proportions of cases with bulging ear drums at the outset were significantly different in the two groups. Also, 43% of the placebo, and 66% of the antibiotic group either had missing data for crying or were not crying at baseline (*p *< 0.001). The Cochrane Review, [42], noted this difference as well, ascribing it to a possible failure of randomization.

(ii) There was direct and indirect evidence that many of the two planned home visits, especially the second one, either did not occur or were appreciably delayed. This affected the collection of historic baseline data, and the delivery of the second parental diary scheduled for the first home visit. It also impacted the weighing of medicine bottles at both visits, and produced high and biased levels of missingness for a key outcome, fever. Fever values were missing for the first home visit for 24% of the antibiotic and 23% of the placebo group; at the next home visit, the respective levels were 55% and 41%, with a significantly smaller proportion of the antibiotic group having data on fever (*p *< 0.001).

Other anomalous patterns of missing data were also observed. At each visit, researchers were to measure the body temperature of the child, to record current pain and weigh the medicine bottles during each visit. For the first home visit, there were 3 missing values for pain, none for analgesic use, but 52 missing values for fever. For the second home visit, the anomalies were even more serious.

The authors only partly consider the problem with the home visits. Thus, in the analysis of consumption of analgesics, they reanalyzed the data to adjust for the "*interval between entry to trial and visit ....*" But no details are given and the adjustment was not done for any other variable. Also, the impact on other aspects of the trial, like delivery of the diaries and identification of treatment failure, was not considered.

The anomalous patterns of missing data for these visits raise the possibility that some of the researcher assessed pain data may have been obtained by telephone, some medicine bottles were later brought to the clinic, and many visits did not actually occur. The rate (and/or timing), notably of the second visit, appear to have been appreciably, if not significantly, higher (and/or earlier) for the placebo group. This scenario puts all home visit data into question. It may have contributed to earlier detection and a higher declared rate of treatment failures in the placebo group. This was a key outcome, for which the issue of timing is critical one (see below).

(iii) One striking feature of the in-clinic visit on day 8 is the paucity of the data given. Of the 16 short term main outcomes, only one (ear drum signs) directly derives from evaluation by the physician at this visit. No data on fever or pain assessment from this visit are given. Evidence indicates that the treatment failures noted at this visit were excluded from analysis, violating the protocol, and possibly skewing the results in favor of antibiotic therapy (see below).

The nature of these problems raises a question about the participation of the 48 trial doctors and other researchers in the training sessions. This factor added to poor conduct during the trial to produce deficient, biased and suspect short term follow up data.

#### Data analysis

Four types of flawed data analytic methods were found: (i) the survival curves for crying were flawed; (ii) some missing data were coded as zero values giving incorrect means and standard deviations; (iii) what were called stratified analyses were actually partial sub-group analyses; and (iv) the intent-to-treat analyses at times used only about 50% of the subjects. Generally, these analytic errors tended to produce results favoring antibiotic therapy. A minor issue was that while all binary data comparisons used risk ratios, they were repeatedly called odds ratios. This particular mistaken identity error rarely occurs in the medical literature [45].

#### Reporting style

The existence and severity of such problems (and others described fully in Additional file 2) can be inferred only by scrutinizing various sections of the paper with great care and connecting different statements and data. For example, baseline data are not tabulated but presented in an unclear narrative manner. Outcomes not favoring antibiotic therapy do not receive due emphasis. Thus, pain was the sole outcome with two distinct sources. Both consistently showed equivalence between the treatment arms. But these results were not only not stressed but dismissed by an explanation that was not consistent with what the authors state elsewhere. That about half of the cases had missing data distributed in a biased manner for fever at the second home visit is nowhere noted even though a significant finding favoring antibiotic therapy was noted in the Abstract. A similar situation pertained to the physician evaluation on day 8, analyses relating to bulging ear drums and other variables.

I examined the analysis of each of the twenty main outcome variables in the light of the biases and methodologic flaws I found. A statistically significant result favoring antibiotic therapy was declared for five of the twenty main outcomes. These were crying, fever at the second home visit, analgesic use, treatment failure and school absence. My reanalysis shows that these results do not hold up to critical scrutiny. Below I summarize my reappraisal for three of these variables.

#### Crying

At baseline, the crying status of roughly 43% of the placebo, and 66% of the antibiotic was either not known or not crying. These data were, however, shown as survival curves indicating "*a real difference in outcome, rather than in characteristic at entry*." Consequently, as compared to placebo, antibiotic therapy was said to lower the mean duration of crying by about a day (*p *< 0.001), a *p*-value smaller than for any other outcome.

But this analysis erroneously put zero as the duration of crying for the cases who either were not crying at baseline or had a missing value when comparing the two groups. In terms of intent-to-treat analysis, this amounts to using the best case scenario for the antibiotic. My estimate from the data on those known to be crying at outset showed that the median duration of crying for both groups was about 1.5 days, and that the pattern of decline in rates of crying in the two groups was quite similar. For crying, the bias at baseline was thus compounded by a flawed and biased analysis. Later, the authors state that some data were reanalyzed to adjust for crying at onset. If all children in both groups (with completed diaries) were crying at outset, as clearly asserted in two earlier parts of the paper, how such an adjustment can be done is not clear.

#### Fever

Fever, an important outcome for AOM, is not noted as a main outcome in the Methods section but is declared as such in Table 1[36]. Fever was not defined, no data on fever at baseline or day 8 clinical visit are given, the home visit fever data are missing at higher levels than for any other variable, and the missingness pattern at the second home visit is highly biased.

While the authors adjusted the analysis of analgesic use up to the second home visit to account for the differing times to the visit, such an adjustment was not done for fever measured at the same visit. The intent-to-treat analysis for fever included only half the randomized subjects. For fever at the second home visit, a significant difference favoring antibiotic therapy was declared. As for crying, data derived from biased conduct were analyzed in a biased manner, yielding a significant finding for fever as well.

#### Treatment failure

2% of the antibiotic group and 14% of the placebo were declared as treatment failures (*p *= 0.001). Several concerns bring this finding, repeatedly stressed in the paper, into question. The first problem was definition: In the Patients and Methods section, treatment failure is defined without a time line. In the Short Term Outcome section, the time line is specified as "*on or before day 8*." The data in Table 1[36], however, exclude the day 8 in clinic visit, and are only for treatment failures "*during*" the first week. In particular, those showing "*clear evidence of clinical deterioration in one or both ears*," as noted by the physician on day at 8, and likely given a second line antibiotic, were excluded. For this latter variable, no significant difference between the two arms was found.

As detailed thoroughly in Additional file 2, varied evidence indicates that this change of protocol reduced the number of treatment failures and induced a bias in the data. Bias in the timing and occurrence of home visits with possibly higher rate or earlier timing of the visits in the placebo groups may have led to a biased pattern and earlier identification of treatment failures. The antibiotic group failures detected later (on day 8) were not counted in Table 1[36]. About 75% of the placebo group treatment failures are vaguely classified as "*Other non-resolution*" (Table 2[36]). Other cases like children withdrawn for severe cough, diarrhea, rash or parental initiative, who may been given a second line antibiotic, also seem not to have been counted. My reanalysis, done to the extent possible from the data given, found a non significant *p *= 0.0933 between the treatment groups. Also, the baseline imbalance between the two arms in terms of bulging ear drums (an indicator for antibiotic therapy in the study) confounded the finding on this outcome.

In a similar manner, the two other outcomes for which statistically significant results were declared, namely, analgesic consumption and absence from school, were affected by biased patterns of home visits and missing data, possible miscoding of missing data values, and lack of baseline comparability.

#### Overall

These problems form a portion of a whole edifice serious and minor problems detailed in Additional file 2. They are not just reporting errors but reflect serious deficiencies and biases in design, conduct and analysis. In the light of these, and the totality of the information in the paper, it is difficult to justify any of the main conclusions drawn. My overall verdict is that Burke et al. is potentially a fatally flawed trial. An independent audit is warranted before any of its data and conclusions can be deemed credible.

### Usage of Burke et al. data

Each systematic review of antibiotic versus placebo for AOM with a meta-analysis has used the data from Burke et al. In many, it was accorded a prominent role in terms the number of outcomes contributed for analyses. I examined its contributions to the three most recent reviews, [42,43,46]. The detailed results are in Additional file 1. Below I give some of the highlights.

Rosenfeld (2003), with a total of nine trials, used the data from Burke et al. in six of the seven meta-analyses comparing antibiotic therapy with placebo or symptomatic therapy. It is one of the three trials for which that was done. Glasziou et al. (2004), the Cochrane Review with ten eligible trials, meta-analyzed eight outcomes. Burke et al. contributed to seven, and in four, it secured the highest weight. Rovers et al. (2006), the sole individual patient data meta-analysis had raw data from six trials, and employed a composite main outcome based on fever and pain at 3 to 7 days. Burke et al. accounted for 14% of the total sample size. The outcomes from Burke et al. used in these three reviews are shown in Table 4 in Additional file 1.

For the outcomes pain and/or fever, each of the three reviews used the researcher based visit data, despite the biased timings and high and biased levels of missing data from the visits. Rovers et al. (2006) misrepresent the researcher based data of Burke et al. as parental diary data. They used visit 3 fever data of Burke et al. in their main composite outcome despite the fact that they were missing (in a biased manner) for about half the cases. It is the only trial in this review where the level of missingness for a key outcome is inordinately high and biased, where no data at all for a key baseline predictor (fever) are available, and for which the data source is wrongly ascribed.

Rosenfeld (2003), the only review to meta-analyze treatment failure, did not note the issue of inconsistent definitions and misidentified outcomes. The Cochrane Review used the data on adverse effects (vomiting, diarrhea and rash) without taking double or triple counting, and the longer time line, into consideration. The data on contralateral pain used have the time line and faulty denominator problem as well. But it did draw attention to a possible failure of randomization in the trial, though the implications were not pursued.

Overall, the three recent meta-analyses used the data from Burke et al. in a manner that was oblivious to the multiplicity of serious problems connected with it. Even when a problem was noted, the data were used anyways. Further, some reviews introduced additional errors. Even Rovers et al. (2006), with access to the raw data of Burke et al., does not note or clarify any of the problems I detected for it.

## Conclusion

I note four limitations of my paper. First, it focused on one study; the other studies in the field need scrutiny as well. [34] assessed a single AOM trial; [6] assessed a single trial in another field. I have gone further than both of these papers in terms of design and methodologic issues. Second, I mainly dealt with internal validity. Evaluation of external validity is crucial [24,25] as well. Three, I was the sole assessor in this endeavor. As a remedy, my findings are given in extensive detail in the two additional files accompanying this paper, so that my own work can be evaluated thoroughly. And, four, my identification of relevant systematic reviews was by a hand search and did not cover languages other than English. The treatment of AOM has, for a long time, been surrounded by controversy, and related trials and reviews have garnered extensive publicity in the medical literature, and at times, in the media as well. Thus, it is unlikely that a relevant review has been missed.

To recapitulate, the check list-based evaluations in the systematic reviews, some by independent and blinded reviewers, and my own check-list evaluation indicated that Burke et al. had the main hallmarks of a good quality trial. Published amidst mounting concerns about the quality of AOM trials, it apparently not only anticipated the call for better quality trials by Claessen et al. [35], but also seemed to have addressed many of the design and other types of weaknesses identified by them. The wide usage of its findings in review papers also indicates the regard accorded to it in the literature. Other review papers and later trials have also referenced it in a positive light (e.g. [27,47]). I have not come across any paper that has raised and documented concerns serious enough to make me question that verdict.

Yet, my detailed dissection revealed a wide chasm between the image and reality. It showed that Burke at al. is a seriously flawed and erroneously reported study. I found good evidence for extreme baseline noncomparability, deficient and biased short term follow up, nonrandom patterns of missing data, erroneous data analysis, and biased interpretation. This sharp contrast between my findings and the earlier evaluations has several implications.

The first relates to the methodology of quality assessment of clinical trials for systematic reviews. Such reviews, especially of definitive and homogeneous trials, stand at the top of the evidentiary hierarchy. They need to be performed in an as thorough and meticulous a manner as that required for a clinical trial. If one after another of the systematic reviews in a field uncritically includes one or more trials of dubious quality and validity, and magnifies the problem by using their data inappropriately, then the basis for regarding systematic review as a gold standard of evidence begins to crumble [48,49].

For one particular issue, antibiotic versus symptomatic therapy for AOM in children, and for one trial, Burke et al., I have shown the existence of such a scenario. That its flaws were not uncovered was due, I hold, not to the particular check list or instrument used but points to a possible shortcoming of the check-list approach to quality evaluation as such.

Quality check lists are useful and essential, and the search for key indicators of quality needs continued attention. However, check lists need to be applied with care, and not as a short-cut. A psychiatrist using a personality scale to form an overall judgment of a patient would be remiss to also not throughly evaluate the patient in person, and in terms of other relevant records. In a similar fashion, each trial in a review needs to be evaluated comprehensively. There is a need to read the report and related papers with care and a singularly critical lens, keeping both subject matter related and general methodologic criteria in mind. Authors should also be contacted for clarification, and, if possible, the study protocol and original data obtained.

While that is a desired ideal, it has to be noted that comprehensive evaluation is a time consuming effort. Though the type and scale of the problems I found for Burke et al. could not have been detected other than by such an evaluation, the extent of the effort involved is indicated by the fact that even a general reading of the paper by a class of post-graduate physicians and seasoned researchers did not bring its serious problems into relief. When these were later explained to them, they were astonished by what they had overlooked. There was, indeed, no short-cut here; all short-cuts led into a blind alley. One referee of my paper aptly described quality evaluation of trials as a daunting task, and likened it to "*a type of detective work that aims to discover in particular what the authors of the trial reports may have wished to conceal*."

Nevertheless, a team performing a systematic review with say, more than twenty trials, cannot be realistically expected to devote the about three person-months I spent to evaluate each trial. I propose three practical steps to at least partly address this dilemma.

### One

A systematic review team needs to include persons who have intimately followed the literature on the topic at hand. Their knowledge would help in the identification of poor or good quality studies that a check-list evaluation may overlook. They may or may not be a part of the group of check-list evaluators. External assistance for this task may also be sought. The basic goal should be to ensure that each included trial report is thoroughly and critically read by at least one person associated with the review.

### Two

In addition to a check-list summary of quality, systematic reviews should provide a two to three paragraph narrative based synopsis of each trial, highlighting its strengths and weakness. This can be posted on an associated web page.

### Three

Systematic review needs to be viewed as a dynamic process. Each review needs a mechanism through which credible concerns about any aspect of the review, including quality evaluation, can be brought to bear into the process. It thereby becomes a dynamic mode of generating knowledge not just in terms of incorporating new studies but also for re-evaluation of studies done in the past. If the authors of the review are obliged to respond to those concerns and undertake needed rectifications, weeding out poor quality studies and including ignored good quality studies would be enhanced. The Cochrane Collaboration has such a mechanism in place; though the extent to which it has functioned effectively in practice needs to be assessed.

The second implications of my paper relates specifically to the trial of Burke et al. I am of the view that the totality of the problems and the extent of bias revealed by my study do suffice to denote it a potentially fatally flawed study. An external audit of all aspects of the trial, and an independent re-analysis of its data are needed. I also hold that including its findings in a meta-analysis before the report of such an inquiry are available is not warranted. My paper, in addition, calls for a detailed evaluation of all placebo-controlled studies of antibiotic treatment of acute otitis media.

Another implication of my paper relates to journal peer review. One goal of peer review is to detect major problems before they become ossified in print. My case study illustrates a failure of this process. Also, no correspondence relating to this trial was published. In the about one and a half decades since this transpired, the issue of trial quality has received greater attention, and journal editors have taken many steps to improve peer-review. Unfortunately, poor quality or deeply flawed trials continue to appear in print, even in the major journals. As the recent startling revelations surrounding several published trials of COX-2 inhibitors in prominent journals show, continued vigilance on that front is in order [50].

The major task, however, is to prevent serious flaws in the design, conduct and reporting of clinical trials. Transparency at all levels is the principal requirement here [51], and registering trials, making protocols available, and instituting a public repository of trial data constitute the basic ingredients of this effort.

In conclusion: "*Reflections on the details of a case [study] allow one to draw broader lessons *..." [52], page 4. Like the case study of [6], my case study has some ramifications beyond its immediate purview. The severe limitations of the check-list approach to quality evaluation for one specific trial I found implies that perhaps there is no short-cut to trial quality evaluation. How often are poor quality trials rated as good quality trials, and does that seriously distort the evidence emerging from systematic reviews? A wider investigation of these questions is required.

## Methods

Two main tasks were undertaken for this study. The first task was to extract the quality assessments of Burke et al. from systematic reviews. The criteria for an eligible review were: One, it covered the comparison of antibiotic therapy with placebo or symptomatic therapy for AOM in children. Two, it was based on primary trials identified by a form of systematic search. Three, its main aim was to summarize the therapeutic evidence. It may or may not have performed a meta-analysis. Four, it included the paper Burke et al. And five, it was published in or before December 2006. Reviews which just summed up the findings from other reviews were excluded. For updated reviews, the most recent version was used. I identified the potential reviews from a hand search of the English literature. Nine systematic reviews published from 1993 to 2006 fulfilling these criteria were selected [37-43,46,53].

From each review, I recorded the quality evaluation method, the quality evaluation for Burke et al., and how its data were used. I focused on issues pertaining to internal validity (selection, performance, detection, attrition, analysis and reporting biases) as broadly described by [16]. The information extracted is detailed in Additional file 1: **Burke et al. (1991) in nine systematic reviews**.

The second main task was to perform an in-depth, comprehensive evaluation of Burke et al. This was done by repeatedly and carefully reading the paper, performing a section by section, and at times, sentence by sentence, dissection, and recording the relevant information. I checked all the data for completeness, consistency and accuracy, and assessed the reasoning, methods and conclusions in various parts of the paper. Where possible, I reanalyzed the data. At times, all potential datasets consistent with other information provided were generated and analyzed. During this process, I kept in mind the same quality components relating to internal validity as those noted above for systematic reviews. Other than that, I did not follow any formal method. Indeed, the approach in such an exercise will necessarily vary from trial to trial, and medical field to medical field.

At the end, I formulated plausible explanations and an overall perspective for the distinct problems I found. Where feasible, flaws of reporting were distinguished from the flaws in the design, conduct and analysis [23]. It took about three months of focused work to complete my in-depth review. The information extracted and the complete picture I formed of this trial are in Additional file 2: **A detailed critique of Burke et al. (1991)**.

To complement these main tasks, two other activities were undertaken. One, I performed a check list based quality assessment of Burke et al. using Table 1 of Balk et al. [19] as the template. This was completed before the in-depth evaluation. The aim, in part, was to compare its conclusions with those I found from the systematic reviews, and in part, to provide an overall, standardized description of the trial for this paper.

The second activity involved a class of medical researchers and postgraduate students attending a course on evidence based medicine conducted by me at the University of Oslo in June 2006. They had had a day of lectures on the history and basic principles of clinical trials in which several examples of poor and high quality trials were given. They were then required to read Burke et al. and another paper (the first reported randomized trial which compared antibiotics with placebo for AOM [44]). At the start of the next class session, they provided an overall quality evaluation of the two papers on a five point scale (1 = very bad, 2 = poor, 3 = acceptable, 4 = good, 5 = excellent). At this stage, the use of specific scales or instruments to assess trial quality had not been discussed. Hence, this was not a check-list based assessment. The aim here was to see whether the students would spot problems with Burke et al. which the systematic reviews had overlooked.

## Competing interests

The author declares that he has no competing  interests.

## Authors' contributions

This study was conceived, designed, executed and written up by the sole author.

## Supplementary Material

Additional file 1 **Burke et al. in nine systematic reviews.** This file gives the details of the quality assessments of, and usage of data from, Burke et al. in nine systematic reviews for antibiotics in AOM. Click here for file

Additional file 2 **A detailed critique of Burke et al. (1991)**. This file provides an in-depth critical analysis of the paper Burke et al. The summarized results given in this paper are derived from this file. Click here for file
