# Supplementary material for: No short-cut in assessing trial quality: a case study
Source: Trials. 2009 Jan 7;10:1. doi: 10.1186/1745-6215-10-1 (PMC2636799; doi:10.1186/1745-6215-10-1)
Supplement: Additional file 1 — Burke et al. in nine systematic reviews. This file gives the details of the quality assessments of, and usage of data from, Burke et al. in nine systematic reviews for antibiotics in AOM. [file 1745-6215-10-1-S1.pdf]

Additional file 1:
Burke et al. (1991) in nine systematic reviews

Karim F Hirji*

Department of Epidemiology and Biostatistics
Muhimbili University of Health and Allied Sciences
P. O. Box 65015, Dar es Salaam, Tanzania

Email: kfhirji@aol.com;

*Corresponding author

Abstract

Background: This **Additional file 1** supplements the main paper Hirji (2009), *No short-cut in assessing trial quality: a case study.* It describes the check-list based quality assessments of the paper Burke et al. (1991) performed in nine systematic reviews of antibiotic treatment for acute otitis media. It also describes the usage of the data from this paper in the three most recent reviews.

Results: Six reviews did not note any specific quality related problem, two reviews each noted one quality related problem, and one review noted two problems. In all three distinct problems were identified: lack of baseline comparability, incomplete description of dropouts, and a possibly misidentified outcome. Most reviews rated the trial as a good or high quality trial, and recent reviews continue to extensively use its outcome data.

Conclusions: Quality assessments done for nine systematic reviews by experts in the field using different checklist based instruments and at times with more than one independent assessor have generally concluded that Burke et al. (1991) is a good or high quality trial.

Quality Assessments

I first relate the quality assessments of Burke et al. (1991) [1], from now on referred to as Burke et al., in nine systematic reviews which looked at antibiotic versus placebo or only symptomatic therapy for acute otitis media (AOM) in children. A broad description of Burke et al. and the criteria used to select the nine reviews appear in Hirji (2009). Below I give the nine quality evaluations in a chronological order.

Systematic Review 1: Lehnert (1993) [2] reviewed five randomized trials and two other studies. Trial quality was evaluated by eleven parameters but no meta-analysis was done. A summary of each trial was provided. In these, critical remarks were made on two of the trials; Burke et al. was not among them.

Systematic Review 2: Rosenfeld et al. (1994) [3] is a multifaceted review. Two independent reviewers blindly evaluated trial quality on an 11 item (0 to 22 points) quality scale derived from [4]. The score variation was summarized but the score for each study was not reported. Some quality concerns were noted. Study selection in this review aimed to ensure that "*only high quality studies evaluating a similar hypothesis*" were combined. Burke et al. was included

in the meta-analysis with four placebo controlled trials; and no specific internal validity related quality concerns for it were raised.

Systematic Review 3: Del Mar et al. (1997) [5] reviewed eight trials. Quality was scored from 0 to 11 points using four basic parameters. Burke et al. obtained 10 points, one of the three highest scoring trials. The score for the paper [6] was 5, not that distinct from the comparative student evaluations reported in Hirji (2009). One low quality trial without patient oriented outcomes was not included in any meta-analysis, and another low quality trial which reported recurrences only was used in just one meta-analysis.

Systematic Review 4: Froom at al. (1997) [7] reviewed seven randomized trials. A wide level of clinical heterogeneity between the trials was noted. No formal meta-analysis was performed. Apart from selection based on key criteria like randomization and double blinding, no formal quality assessment was done, and no specific concerns for any trial were raised.

Systematic Review 5: Cantekin (1998) [8] was a multifaceted review. It did not have a formal quality review, or a meta-analysis. Critical comments were given only for the studies favoring antibiotic therapy. On antibiotics for AOM, eight placebo controlled trials and five other studies were examined. The findings of Burke et al. were queried due to "*the entry imbalance of symptoms between the treatment groups,*" notably in terms of crying. Adjusting for such imbalances would, it was stated, make the short term improvement in the two groups equivalent. It was also noted that this trial was "*financed by a drug company (SmithKline Beecham) with probably the largest market share of the otitis media market.* "

Systematic Review 6: Marcy et al. (2001) [9] was a multifaceted review. Trial quality was judged on the 0 to 5 points Jadad scale [10] by two independent reviewers. Discord was resolved by conference. Burke et al. was included in the six trials based comparison of ampicillin or amoxycillin with nonantibiotic therapy. With the quality score of 4 out of 5, it was one of the five trials selected for meta-analysis. The sole issue raised for it was an inadequate description of withdrawals and dropouts. But no details were given.

Systematic Review 7: Glasziou et al. (2004) [11] is the current Cochrane Review with ten eligible studies. A modified version of the quality assessment scheme of [12] was employed by blinded independent reviewers. Discord was resolved by discussion. Scores for each trial were not reported but we read that "*[t]he methodologic quality of the ten eligible studies was generally high.*"

In the section on Characteristics of included studies, Burke et al. was noted as a double blind study which used medicine bottles with sealed randomization code, and was given a high score (A) for concealment of allocation. Two critical comments for it were: (i) The amoxycillin group seems to have had fewer children crying at baseline, and (ii) an outcome entry in Table I (occurrence of discharging ears) of Burke et al. was not clearly defined. The first problem, also noted in [8], was ascribed to a possible "*failure of randomisation.*" The issue of not including all children in follow up assessments was raised for two trials, but not for Burke et al.

Systematic Review 8: Rosenfeld (2003) [13] was a multi-faceted review. Nine trials were included for comparing antibiotic versus nonantibiotic therapy for AOM. Quality was assessed in terms of three basic criteria. The trials were "*generally of high quality …,*" the one exception being a trial that was not blinded. Burke et al., we note, was a double blind study.

This review has a long quote from Burke et al., a part of which said: "*…children included in the study did not represent a cross section of all those with acute earache, but were selected on the basis that treatment with placebo would pose no ethical problems, and their inclusion was subject to informed parental consent.*" That this trial violated its own ethical standard by recruiting children with bulging ear drums was not indicated.

Systematic Review 9: Rovers et al. (2006) [14] is the first individual patient data meta-analysis (IPDM) of antibiotic versus symptomatic treatment for AOM. Six trials with available data were included, and judged under four major quality criteria. The raw data for each study were "*thoroughly checked for consistency, plausibility, integrity of randomization, and follow-up. A few issues were queried with the responsible trial investigator or statistician, and all were resolved.*" The quality scores by trial were not given but the quality of the six trials was "*generally high.*" It was also noted that the loss to follow up for all studies was less than 10%.

Some of these reviews also raised issues relating to external validity, some of which applied to Burke et al. These included exclusion of young chil-

dren, reliability of criteria for diagnosis and outcome, exclusion of children with severe AOM, and so on ([2, 7, 9, 13]).

In sum, in terms of internal validity, only three of the nine reviews noted at most one or two concerns with this trial. These included a possible absence of comparability of groups (crying at baseline), an unclear outcome variable, and some problem with follow up. But these were noted in isolation, and not explained, and with the exception of one review, the trial was nevertheless deemed overall as a good quality trial. The one highly critical review examined only one issue, and that in a superficial and apparently biased manner.

Usage of Data

The first systematic review of antibiotic versus placebo for AOM used the data from Burke et al. for its main meta-analysis (the actual outcome is not clear), and denoted its results on crying, fever and school absence as providing further evidence in favor of antibiotic therapy [3]. Since then, each systematic review in this field which has included a formal meta-analysis has generously used the data from this study in one or more meta-analyses.

We consider the situation in the three most recent systematic reviews. : (i) [13] used the data from Burke et al. in six of the seven meta-analyses for comparing antibiotic therapy with placebo or symptomatic therapy. It is one of the three trials (out of nine) for which that was done. (ii) Eight outcomes were meta-analyzed in the current Cochrane Review, [11]. Burke et al. contributed to seven. And in four, it was accorded the highest weight. (iii) The main outcome in the individual patient data meta-analysis [14] is a composite outcome based on fever and pain at 3 to 7 days; the relevant raw data from Burke et al. were used in this context.

The specific outcomes from Burke et al. used in these three reviews are shown in Table 4.

For the outcome(s) pain and/or fever, each review used the researcher based visit data. The biased timings of these visits and relevant missing data were not noted. [14] misrepresent the researcher collected data in Burke et al. as parental diary data. Thus, it is incorrectly stated that fever was recorded by parents. And as it is not clarified that pain assessments were done by both parents and researchers, it seems that even for pain they used researcher data (but called it parental data). The list of the outcomes of Burke at al. is also incomplete (see Table 1 of [14]).

[13] meta-analyzed symptom relief at three time points: by 24 hours, 2 to 3 days, and 4 to 7 days, and used visit 2 and visit 3 data from Burke et al. for the first and third time points. For another study, [15], data for all the three time points were extracted from a parent score card based graph. Why a similar thing was not done for Burke et al., and the middle time point was left out, is not clear.

The visit 3 fever data in Burke et al. were missing (in a biased manner) for about half the cases. Yet, they were used in [14] as a constituent of the main outcome. The extent of missingness may have been masked by the use of a composite outcome (pain, fever or both). The statement in this review that the loss to follow up rate in the six included studies was less than 10% is thus somewhat misleading. Burke et al. is the only trial in the review with a high and biased level of missingness for a key outcome (fever), zero data for a baseline predictor (fever), and for which the data source is wrongly ascribed.

[13] is the only review which meta-analyzes the outcome treatment failure. Only two trials contribute to this outcome: Burke et al. and [15]. The definition of treatment failure is different in the two studies. In Burke et al., it is inconsistently defined in terms of the time line, and relevant cases seem to have been left out (see Additional File 2 (bkdc.pdf) for an elaboration). All these issues were ignored.

On perforations, the Cochrane Review used numbers from Table II of Burke et al. Yet, its summary for the study noted the possibility of more perforations than indicated here. Why it then used the data it itself deemed to be of doubtful accuracy is not clear. The variable "occurrence of discharging ears" (Table I of Burke et al.) could have also been chosen. Though this has the limitation that it was noted over a 21 day period by parents and may have included some nonsevere cases as well. The number of treatment failures may have been somewhere between what was indicated by these two variables. The one and three month tympanometry data from Table I of Burke et al. as used by [13] and the Cochrane Review [11] did not incorporate the minor errors we noted; these hardly affect the results; the point is that the correction just required a simple check.

The data on adverse effects (vomiting, diarrhea and rash) from Burke et al. were used in the

Cochrane Review [11] and [14]. The former added the count for each effect to get the total count for a single composite outcome. This possibly resulted in double or even triple counting, as some cases may have had two or all of the adverse effects. Further, unlike other studies, these numbers were compiled over much longer (21 day) period. Burke et al. then secured a high weight in this meta-analysis, skewing the estimate of the summary measure. [14] analyzed each adverse effect separately, avoiding the multiple counting problem. But the issues of the longer time window and higher subsequent counts remained. [13] did not meta-analyze adverse effects.

For contralateral pain, the Cochrane Review used data from Burke et al. that were recorded over a three week period by parents. The denominator used are not accurate, as they do not reflect the missing diaries among the cases with unilateral otitis at the outset. The other issues is: If parental data are used here, why also not use parental diary data for the other two pain outcomes?

For crying at outset, the Cochrane Review ascribed the observed pattern to a **failure of randomization**. What does that mean? Was the randomization process flawed? Why was the failure manifested at such a serious level? Instead of clarifying the issue, the possibly flawed study data are used anyways.

One basic aim of [14] was to identify relevant baseline factors that could guide the choice of initial antibiotic therapy in practice. The seventeen baseline factors noted are in Table 2 of [14]. Burke et al.: (i) had no data six (siblings, being breastfed, passive smoking, coughing, runny nose, fever). Indeed, the number of missing cases on fever at baseline exactly equals the sample size of Burke et al.; (ii) had all cases with a "yes" value for two factors (winter season, ear pain) and all cases with a "no" value for three factors (age < 2 year, otorrhea, perforation), (iii) had unreliable data on two factors (crying and bulging ear drums). (The latter, unlike for other studies, was to have been an exclusion criterion); and (iv) had relevant data only for four factors (sex, recurrent AOM, laterality, red tympanic membrane).

The specific independent predictors of enhanced benefit of antibiotic treatment used were age < 2 year, fever, bilateral AOM, and concurrent otorrhea. For these factors, Burke et al. has no data on fever, is completely confounded in terms of age and otorrhea, and thus contributed meaningfully only in terms of laterality.

To reduce the complexity and bias resulting from deletion of cases due to missing data, [14] employed multiple imputation to fill in the missing values for the outcomes and factors. As noted in Hirji (2009) and Additional File 2 (bkdc.pdf), Burke et al. had a high level of data collection bias for key outcomes, a biased pattern of missing data for some baseline factors, was as an entity confounded with several such factors, and had other serious problems in its implementation. In particular, there were no baseline data, and only about 50% of the outcome data, for fever. In such a situation, imputation is not an appropriate remedy as it only spreads the errors and biases of this trial to the entire data set.

In sum, the three most recent relevant meta-analyses have used the data from Burke et al. in a manner oblivious to the multiplicity of serious problems we have noted. Even when a problem was noted, the suspect data were used anyways. Also, some reviews added their own errors. [14] had the raw data for each included study. They were said to have been checked for errors and consistency done. Yet, it too did not clarify any of the problems we noted for Burke et al. In fact, it misrepresented the nature of the outcomes it used from this study.

The continued uncritical use of data from this trial not only pushes the multiplicity of serious problem we have noted from this trial under the rug but also compromises the validity and accuracy of the conclusions drawn in the systematic reviews.

References

1. Burke P, Bain J, Robinson D, Dunleavey J: **Acute red ear in children: Controlled trial of non-antibiotic treatment in general practice**. *British Medical Journal* 1991, **303**:558–562.
2. Lehnert T: **Acute otitis media in children: Role of antibiotic therapy**. *Canadian Family Physician* 1993, **39**:2157–2162.
3. Rosenfeld RM, Vertrees JE, Carr J, Cippole RJ, Uden DL, Giebnik GS, Canafax DM: **Clinical efficacy of antimicrobial drugs for acute otitis media: Meta-analysis of 5400 children from thirty-three randomized trials**. *Journal of Pediatrics* 1994, **124**:355–367.
4. Marchant C, Shurin PA: **Antibacterial therapy for acute otitis media: A critical analysis**. *Review of Infectious Diseases* 1982, **4**:506–513.
5. Del Mar C, Glasziou P, Hayem M: **Are antibiotics indicated as initial treatment for children with acute otitis media? A meta-analysis**. *British Medical Journal* 1997, **314**:1326–1329.

6. Halsted C, Lepow ML, Balassanian N, Emmerich J, Wolinsky EJ: **Otitis media: Microbiology and evaluation of therapy**. *Annals of New York Academy of Sciences* 1967, **145**:372–378.

7. Froom J, Culpepper L, Jacobs M, DeMelker RA, Green LA, van Buchem L, Grob P, Heeren T: **Antimicrobials for acute otitis media? A review from the International Primary Care Network**. *British Medical Journal* 1997, **315**:98–102.

8. Cantekin EI: **Aggressive and ineffective therapy for otitis media**. *Otorhinolaryngol Nova* 1998, **8**:136–147.

9. Marcy M, Takata G, Chan LS, et al: **Management of Acute Otitis Media**. *AHRQ Evidence Report/Technology Assessment* 2001, **15**:?? [Agency for Healthcare Research and Quality Publication No. 01-E101, Rockville, MD].

10. Jadad AR, Moore RA, Carroll D, Jenkinson C, Reynolds DG, Gavaghan DJ, McQuay HJ: **Assessing quality reports of randomized trials: Is blinding necessary?** *Controlled Clinical Trials* 1996, **17**:1–12.

11. Glasziou PP, Del Mar CB, Sanders SL, Hayem M: **Antibiotics for acute otitis media in children**. *The Cochrane Database of Systematic Reviews* 2004, **Art. No: CD000219.pub2**. [Doi: 10.1002/14651858.CD000219.pub1].

12. Chalmers I, Adams M, Dickersin K, Hetherington J, Tarnow-Modi M, Meinert C: **A cohort study of summary reports of controlled trials**. *Journal of the American Medical Association* 1990, **263**:1401–1403.

13. Rosenfeld RM: **Clinical efficacy of medical therapy**. In *Evidence-Based Otitis Media*, 2nd edition. Edited by Rosenfeld RM, Bluestone CD, Hamilton & London: BC Decker Inc. 2003:199–226.

14. Rovers MM, Glasziou P, Appleman CL, Burke P, McCormick RA, Damoiseaux RA, Gaboury I, Little P, Hoes AW: **Antibiotics for acute otitis media: A meta-analysis with individual patient data**. *Lancet* 2006, **368**:1429–1435.

15. Thalin A, Densert O, Larsson A, Lyden E, Ripa T: **Is penicillin necessary in the treatment of acute otitis media?** In *Proceedings of the International Conference on Acute and Secretory Otitis Media*. Edited by ??, Amsterdam, The Netherlands: Kegler Publications 1986:441–446.

Table 4 - Usage of data from Burke et al. (1991) in three recent AOM systematic reviews

	Review		
Outcome	Rosenfeld (2003) [13]	Glasziou et al. (2004) [11]	Rovers et al. (2006) [14]
Pain Visit 2	***	***	
Pain Visit 3	***	***	***
Contralateral Pain		***	
Fever Visit 3			***
Treatment Failure	***		
Adverse Effects		***	***
Complications†	***		
Perforations		***	
Effusion 1 month	***	***	
Effusion 3 months	***	***	

Note: † mastoiditis or meningitis; *** denotes a used outcome.
