# Supplementary material for: No short-cut in assessing trial quality: a case study
Source: Trials. 2009 Jan 7;10:1. doi: 10.1186/1745-6215-10-1 (PMC2636799; doi:10.1186/1745-6215-10-1)
Supplement: Additional file 2 — A detailed critique of Burke et al. (1991). This file provides an in-depth critical analysis of the paper Burke et al. The summarized results given in this paper are derived from this file. [file 1745-6215-10-1-S2.pdf]

Additional file 2:
A detailed critique of Burke et al. (1991)

Karim F Hirji*

Department of Epidemiology and Biostatistics
Muhimbili University of Health and Allied Sciences
P. O. Box 35015, Dar es Salaam, Tanzania

Email: kfhirji@aol.com;

*Corresponding author

Abstract

Background: This is **Additional file 2** for the main paper Hirji (2009), *No short-cut in assessing trial quality: a case study.* It gives a detailed dissection and quality assessment of the report by Burke at al. (1991) of a clinical trial of antibiotic treatment of acute otitis media (AOM) in children.

Results: The trial in Burke et al. (1991) had many serious flaws in its design, conduct, analysis and reporting. A summary of these flaws is given in Hirji (2009).

Conclusions: The extent and severity of these flaws, in our view, suffice to denote it a potentially **fatally flawed study**. An independent audit of the trial, including a reanalysis of its data, are essential before the findings of this study are used in a systematic review.

Burke at al. (1991) [1] compared amoxycillin with placebo for mild acute otitis media in children. A description of the trial, from now on referred to as Burke et al., appears in Hirji (2009). The quality of this trial has been assessed in nine systematic reviews. It was further subjected to a check list based assessment by the author and an overall student evaluation. The verdict emerging from all these evaluations is that it has, in the main, the features of a good quality trial (Hirji 2009).

This file presents a meticulous evaluation of this trial based on a section by section, item by item, and in places, sentence by sentence, dissection of the paper. My aim was to thoroughly assess the design, conduct, data analysis and report of this trial. After identifying the specific problems, I also undertook the task of linking them up to better understand their sources, and create an overall narrative of what possibly went wrong in the trial.

Results

The problems identified by this detailed evaluation are presented below under the following headings: short term outcomes, sample size, eligibility criteria, baseline comparability, crying, pain, short term follow up and missing data, fever, treatment failure, bulging ear drums, medium and long term outcomes, statistical analysis and presentation style.

Roman numbered tables and figures refer to the tables and figures in Burke et al. The tables for this file are labeled from Table 5 to Table 12.

Short Term Outcomes

Burke et al. does not have a primary outcome. Instead it has a series of short term, two medium term, and two long term main outcomes. At times, the paper refers to them as "*main outcome measures*" (Abstract), and at times, as "*principal outcome measures*" (Table I). I only use the former label.

The outcomes are declared or reported as main outcomes in three places. But that is not done in a consistent manner (Table 5). Complications of treatment, for instance, are absent from the Abstract. Ear drum signs is named a main outcome in the Abstract but not reported there, while fever is not so declared but is reported there. To get a count of the short term outcomes, I added the eight binary and five continuous short term outcomes of Table I to the three complications related outcomes (vomiting, diarrhea and rash) from the last paragraph of the Short Term Outcome section. This gave a total of sixteen main short term outcomes, and twenty main outcomes altogether.

Eight of the 16 short term outcomes were recorded by parents, six by researchers during home visits, and one (ear drum signs) by general practitioners on the day 8 clinical visit. There is one key outcome (failure of treatment) for which the recorder, identifier and timing are not clear. Why only one outcome is reported from the day 8 clinical visit and evaluation is somewhat intriguing. In particular, why are there no data on fever or pain from this visit?

The definition of some outcomes is a concern. Fever is not defined, clinical signs (Patients and Methods) is narrowed down to ear drum signs (Abstract and Table I), and, as I show later, there are two different time lines for treatment failure. The short term outcomes include some outcomes noted at 24 hours and some evaluated for upto 21 days. For AOM trials, that type of grouping is somewhat unusual.

The implications, in terms of validity and precision, for a study with 232 cases to not have a primary outcome but have **twenty** unevenly specified main outcomes are pursued below.

Sample Size

The sample size computations of Burke et al. were for a two group, one binary outcome trial with 80% power to detect an effect. The specific outcome was not stated. It is noted later in the Discussion that: "*The number of children included had been estimated at the outset as sufficient for analysis of common complications, such as effusion of the middle ear; ...*" (para 3). But this refers to a medium term outcome while the focus and major rationale for the study related to short term outcomes.

Using the values, $\pi_1 = 0.15, \pi_2 = 0.30, \alpha = 0.05$, and $\beta = 0.8$, the authors declare that: "*some 200 patients would need to be recruited*" (Patients and Methods, para 1). Applying these values in the usual formula

$$n \;=\; (z_{\alpha/2} + z_\beta)^2 \times \frac{\pi_1(1-\pi_1) + \pi_2(1-\pi_2)}{(\pi_1 - \pi_2)^2}$$

the required n per group is 120, or a total size of 240. For the attained size of 232 children, the stated number 200 gives the incorrect impression that more subjects than needed were recruited, and that an allowance for missing data and loss to follow up was made.

At 90% power and 10% allowance for data loss, the needed total sample size is at least 350. Accounting for twenty main outcomes would raise that number. Further, many baseline factors and outcomes had missing data, two key outcomes had high levels of missing data, and 12% of the cases were included in violation of a key eligibility criterion (see below). The adequacy of the sample size attained is then further subject to question.

In the Discussion, the authors recognize the need to adjust for multiplicity. But they did not perform such an adjustment. I discuss this matter later.

Eligibility Criteria

The declared study population of Burke et al. was children 3 to 10 years old with **mild** otitis media (our emphasis), defined as acute earache and at least one abnormal ear drum. Cases "*for whom antibiotics were thought to be strongly indicated*" were to be excluded (Patients and Methods, para 4). The noted indicators for antibiotic were bulging ear drum (BED), perforation or pus, severe illness and grommets (Introduction, para 3 & Results, para 1; see [2], Evidence Table 2). The form used to assess eligibility, Figure 1, thus has an item on the status of the ear drums.

Accordingly, 27 children (all from one practice) with one or two BEDs at the initial visit were excluded. But this procedure was not followed uniformly. We read later: "*The two groups had similar*

physical signs at entry, except that 19 children in the antibiotic group had one or more bulging ear drums compared with eight in the placebo group." (Characteristics of Children, para 1). Twenty seven (19 plus 8) such cases were included and randomized. Is the equality of these exclusion and inclusion numbers a coincidence, or, were they the same cases counted twice due to data management and programming errors?

Reliable information about excluded children was available from only **one** of the **seventeen** practices (Results, para 1). This was the one that excluded the 27 cases with BED. The sixteen other practices either did not fill out, misplaced or did not return the eligibility assessment form for some or all of the potentially eligible cases. The failure to keep adequate records and recruitment of ineligible cases raises the query: How many of the 48 general practitioners actually attended the training workshops? Were cases with other indications for antibiotic, like perforation or pus, also possibly recruited into study, but the fact not known due to inadequate records?

Another exclusion criterion was entry into the study in the previous 12 months. As this was a three year study, some children may have been included more than once. If so, how many?

The authors later state that study subjects "*were selected on the basis that treatment with placebo would pose no ethical problems.*" (Discussion, para 2). Burke et al. has accordingly been singled out for praise in a review [3]. Yet, at least 12% of the children were recruited in a violation of the scientific and ethical standards set by the investigators themselves. This has not been noted in any systematic review thus far. We examine how these cases were treated in data analysis later on.

Baseline Comparability

RCT reports generally need a table showing the comparability or otherwise of the randomized groups in terms of relevant features. Burke et al. does not have such a table; instead the information is conveyed in a narrative form in paragraph 2 of the Results. The reporting style in this paragraph varies from sentence to sentence. Full comparative data are given only for gender; for two other binary variables, only the group numerators are given; for one continuous variable, only the group means are stated. For the seven other variables, no specific comparative data are provided.

No p-value for any comparison is given. I synthesized the relevant data from this paragraph and show it in Table 6. Where possible, using information from other sections of the paper, I computed the p-value.

In this context, Burke et al. uses term 'significant difference' for only one variable (duration of pain before entry). For the rest, the terminology is unclear. Thus, for a variable declared as showing a difference – gender – the p-value I computed is not statistically significant, while for another declared as not similar – bulging ear drums – it is statistically significant.

No comparative information is given for key baseline characteristics like laterality, crying, fever, cough and body weight. A careful perusal of the paper is needed to locate some of this information. Let us consider what is available in that respect.

For laterality, we find that 98 antibiotic and 102 placebo group cases had unilateral otitis at presentation (Short Term Outcome, para 1; item 7 in Table I; Table III). If no data were missing data for the antibiotic group, then **exactly 16** cases in each group had initial bilateral otitis. The comparison chisquare p-value was equal to 0.9163.

For crying, assuming no missing data, the proportions in that state at presentation are estimated from Figure 2. For the placebo group, it is roughly 57% (n = 113) and for antibiotic group, it is about 34% (n = 107). The difference has a high statistical significant ($p < 0.001$), and remains so even if somewhat different estimates are drawn from the figure.

The authors explicitly (but later in the text) declare that no baseline differences with respect to crying existed. "*Note that as figure 1* [sic] *is a survival curve, the differences between the groups at time 0 represent a real difference in outcome, rather than in characteristic at entry*" (Short Term Outcome, para 1). On the other hand, two systematic reviews have pointedly declared it a baseline difference [4, 5]. I examine this issue in depth in the next subsection.

The baseline data for fever do not appear anywhere. It is only stated that, except for bulging ear drums, both groups were similar at the outset in terms of physical signs. (The group-wise data on fever at visit 2 and visit 3 are noted in Table I, but the fever data for visit 4 are also not given anywhere.)

For the outcome analgesic consumption, the authors reanalyzed the data to adjust for body weight; a hint that the weight distribution by group was dif-

ferent enough to warrant it. But no specific data are provided. There are no overall and group-wise data on cough at presentation as well.

Some other concerns are: If children with perforation or pus at presentation were (in error, like for BED) randomized, how were they distributed? Was randomization blocked or stratified by practice? If not, how were the antibiotic and placebo cases distributed by practice? Did some have mostly placebo children and some mostly antibiotic children? We only know that one of the seventeen practices accounted for 36% of the children recruited.

The description of baseline comparability is thereby not satisfactory. Relevant data are absent for most factors, important factors are ignored, key terms are unclearly employed, and the reader has to perform the computations to decode them. The factor for which (going by the authors' data and interpretation) we can state that the groups did differ in a statistically significant fashion was a critical factor, namely, bulging ear drums. According to the authors, it was not only predictive of response to treatment, but also that cases presenting with BED were not to be included in the study. For crying, something unusual may have occurred, as the initial difference between the groups is too large to be ascribed to chance variation.

The above noted concerns indicate that the recruitment and randomization stages of the study may have faced hitherto unrevealed obstacles. I hypothesize that the omission of important baseline data may be connected to (i) deficient training for the investigators and (ii) inadequate and biased short term follow up (see below).

Crying

Now I examine the data on key outcomes, starting with crying. With the exception of the situation at time 0, the crying data came from parental diaries only. The stated results show a sharp difference between the two treatment groups, with a p-value smaller than for all the other variables in Table I. The authors note these results in the Abstract, explain the crying curves of Figure 2 in two places, and relate these findings in the Short Term Outcome subsection and the Discussion.

The Cochrane Review critiques the authors' survival curve interpretation of the data on crying thus: "*Figure 2 appears to show that, at baseline (0 hours), fewer children were crying in the amoxycillin arm, suggesting a failure of randomization.*" [5]. Let us explore this matter.

Survival curves start at 100%. If the Figure 2 curves for crying are survival curves, as the authors declare, then all children (with completed diary data) in both groups were crying at time 0. It further implies that soon afterwards only about 57% of the placebo, and about 34% of the antibiotic, children were in that state. The duration of crying (from time 0) for 43% of the placebo group, and 66% antibiotic group was then zero or almost zero, and so the sharp drop in the survival curves. Such an instantaneous and differential effect is not biologically plausible. Nowhere do the authors directly say if that was what they observed.

The Cochrane Review implies that about 43% of the placebo, and 66% antibiotic group children were not crying at the time of entry. There are three other reasons to support this. (i) Burke et al. performed an analysis adjusting for several factors including crying at onset (Long Term Outcome, para 2). If all children were crying at the start, an adjusted analysis is not relevant, if not impossible. What the authors say here thus contradicts their previous survival curve explanation. (ii) The sharp difference in crying is not consistent with the finding that at all stages of the study children were in pain (noted by both parents and researchers) at very similar levels in the two groups. (iii) If there was indeed a sharp initial drop, it would be recorded at the next observation point, namely four hours after start, and the crying curves would drop at that point rather than at the zero time point.

Discarding the authors' explanation implies that comparing crying duration is valid only for cases crying at time 0. Therefore the mean durations of crying stated in the paper **are definitely incorrect** as they include the cases not crying at the outset. Further, since such cases were unevenly distributed between the treatment groups (chisquare $p < 0.001$), all the analysis done for crying in Burke et al. is subject to **a strong bias**.

The basic question is: how was it that such a biased analysis was first performed and then strongly defended in the paper? I think the confusion arose as follows: There was no systematic procedure for recording whether a child was crying or not at the initial visit. It was explicitly noted only for some cases. Accordingly, 57% of the placebo group children and 34% antibiotic group children were recorded as crying at time 0. The remaining

cases (not crying or crying status not known) were recorded using such a code that a missing datum was deemed a valid zero value by the statistical software used. It then included these values to compute and print the survival curves. With a computer print out in hand, the authors could make the forthright assertion that the curves showed "*a real difference in outcome, rather than in characteristic at entry.*" This assertion is but an outcome of a real absence of communication between the data analyst and clinical researchers (in addition to other reasons we consider later) than of any real result of the study.

Random variation is an unlikely sole source of the marked baseline difference for crying. The possibility of experimenter bias cannot be discounted. We note that significantly more data for fever (also a key outcome) at visit 2 and visit 3 were available for the placebo than for the antibiotic group, skewing the analysis for fever as well (see below). The manner of randomization may have been a factor too. If it was not stratified by practice, the practices with poor data management may have been overrepresented in one of the treatment groups. (A vast majority of the practices are known to have not kept at least some of the records well).

Whatever the explanation, the analysis of crying in Burke et al. is **plainly incorrect**. Assuming no bias, and with available data, a conceptually more valid, yet crude, arbitrary time point based analysis of crying is done as follows. We look at the 24 hour **cessation of crying among those crying at time 0.** At time 0, about 64 (57% of 113) placebo group and 35 (33% of the 106) antibiotic group cases were crying. At 24 hours, these numbers are respectively estimated to be 19 (18% of 35) and 45 (40% of 64). The twenty four hour crying stoppage rates are 30% for the placebo, and 46% for the antibiotic group, with a chisquare p-value = 0.1108, RR = 1.54, and 95% CI for RR being (0.90,2.58). This is within the usual realm of chance variation. We compute the correct mean durations of crying among those known to be crying at the start using the formula

$$\frac{n_*\bar{x}_* + (n - n_*) \times 0}{n} = \bar{x}$$

where $\bar{x}$ is the given mean, $\bar{x}_*$ is the adjusted value, n is the group sample size, and n_* is the number known to be crying at the start in the group. Then the mean durations of crying for placebo and antibiotic groups are about 2.5 and about 1.5 days, respectively. The median duration of crying for those initially crying, estimated from Figure 2, is about 1.5 days for each group. However, this partial reanalysis does not take us far, remains biased, and nothing definitive can be said so long as the questions posed above remain unanswered. (Note, the use of inappropriate zero values as done for crying may apply to other variables like analgesic use as well (see below).)

Pain

Pain was the sole outcome assessed in two distinct ways, by the researchers and parents. The former evaluated pain at the initial visit and during two home visits, and the parents recorded pain in the 24 hour diary and the 21 day diary. Items one and three in Table I (pain at visit 2 and pain at visit 3) derive from the former source. Item nine (duration of pain), item seven (contralateral pain), and the curves for pain in Figure 2 derive from parental diaries. Note, the parental diary records began four hours after the initial visit.

Since acute earache was a key inclusion criterion, it is safe to assume, even in the absence of an explicit record, that each child was in pain at time 0. In the light of the discussion on crying, even such a mundane point has to be noted. Accordingly, there is no problem with calling the pain related curves in Figure 2, survival curves. A minor issue is that subjects with incomplete data on pain could have been used in the survival analysis, increasing the effective sample sizes for this variable.

The diary data were not available for 12 of the 232 cases. The missingness levels for pain data from the researcher visits seem smaller (3 for visit 2 and 7 for visit 3). But, as we argue later, the actuality, timing and nature of these visits are subject to question.

The results showed a clear difference between researcher and diary based data in absolute perception of pain. At 24 hours, parental reports noted about 70% of the children in each group still in pain (Figure 2), while researchers at visit 2 reported slightly less than half of the cases in each group in pain (Table I). Nevertheless, for comparing the two groups, the relevant p-values and CIs from Table I, and the analysis of Figure 2 data provide a common message: that the observed differences could well be due to random variation. In terms of comparing the effect of treatment, parental diaries were wholly consistent

with direct observations by the researchers.

The authors do note this consistency (Short Term Outcome, para 1). Yet, later they give an explanation contradicts it: "*Diary records of pain were discordant with other measures of short term outcome in this study such as consumption of analgesics and crying. We suggest that such diaries are not a highly valid measure of outcome in children and this may help to account for the negative outcome in previous studies.*" (Discussion, para 5).

This explanation overlooks the fact that for comparing pain, the diary said what the researcher observed. And that the results for crying, repeatedly noted in the paper, were mostly from the *parental diaries* is circumvented. When differences are significant, as for crying and absence from school, diary records seem to be valid. The data on contralateral pain and the occurrence of discharging ears were also based on the 21 day diary. But they did not elicit such a dismissive comment. The basis of the confusion with the data on crying was the paucity of the baseline clinical records. The data on consumption of analgesics, as measured by the researchers, were also unreliable and possibly biased (see below). It was the researcher measured outcomes, which they uphold, that were thereby not as much reliable. Their explanation for pain in the Discussion thereby is in stark contrast to the other information given and noted by themselves. This is one example among several of biased interpretation of the study findings in this paper.

Later I argue that the visit based pain data in part may actually have been obtained by telephone, and are also tainted by the timing bias associated with the visits. I note that the authors of Burke et al. have permitted the diary data on pain from their study to be used in a recent individual patient data meta-analysis [6]. We may wonder what has changed to make these data valid and reliable now?

Short Term Follow Up and Missing Data

Table 7 summarizes the level of follow up in the various phases of the study. For now, consider the short term phase. 92% of the cases were seen at the clinic on day 8, and 95% of parental diaries were returned. This seems to be acceptable at first blush. But these numbers do not reflect other serious problems with the short term follow up.

The first clue is provided by the missing values for the short term outcomes, shown in Table 8. Consider the data on fever: at visit 2, 24% of the antibiotic, and 23% of the placebo group values were missing; at visit 3, the respective missing values were as high as 55% and 41%. The authors clearly say that during the home visit, the researcher was to measure the body temperature of the child, to record current pain and weigh the medicine bottles (Patients and Methods, para 6). The paradox then is why, for instance, for visit 2, there are only 3 missing values for pain and none for analgesic use, but 52 missing values for fever? The visit 3 rates of missing data for fever are much higher and significantly unequal in that a smaller proportion of the antibiotic group children have the data available (p-value < 0.001). (Note the missing or unreported levels for the data on fever for visit 1 (baseline) and visit 4 (in doctor's office) were both 100%!)

Many queries arise: Were the researchers sloppy? Was the child not at home? Did all home visits actually take place? Was some information obtained by telephone? If a visit took place but the child was at school, was it taken for granted that he or she was no longer in pain? In that case, why not also assume that he or she had no fever? All values for analgesic use upto visit 2 were complete; if all home visits did not take place, did some parents later bring the bottles to the clinic for weighing? Or were some collected later?

We have indirect evidence, at least for visit 3, that it did not occur in a uniform way. For, in relation to consumption of analgesics, the data were reanalyzed to adjust for, "*interval between entry to trial and visit [3]*" (Short Term Outcome, para 2), implying that the intervals were possibly significantly different between the two groups.

How different were the timings for these visits? Which group was generally visited earlier? And why? Did a higher proportion of home visits actually occur for the placebo group? How did the differential follow up impact key study parameters like identification of treatment failures? If the about a quarter of missing data for fever at visit 2 is indicative of delayed or nonoccurrent visits, then the delivery of the 21 day diary to the parents of the affected cases was also delayed. Did that impact the completeness and accuracy of the diary records? Not a single detail on these issues is provided.

For fever, one possibility is that it was not a design variable and some researchers recorded it and others did not. Thus the high level of missing data for this variable. But that does not explain the

significant data collection differences between two blinded groups, and is inconsistent with the explicit statement that the researchers were to record body temperature during the home visits.

Now consider contralateral pain (Table 8, item 7). These data were derived from the 21 day parental diaries (given to the parents by the researcher during visit 2), and apply to the cases with unilateral otitis at outset. There are no missing values for this variable. Since completed diaries were obtained for 107 amoxycillin and 113 placebo cases, and all the cases with unilateral otitis had such diary derived data, it implies that only the cases with bilateral otitis at the outset had incomplete 21 day diaries. How does one explain this strong association between laterality and the completeness of a diary?

Consider next the outcome absence from school; this applies to children of age 6 years or more. The data were also extracted from the 21 day diary. Absences from school were not recorded in the first year (1986-87) of the study. Why not? We do not know. Was the diary format modified in the second and third year to include this item? Whatever the case, this may be why absence from school is not a main or principal outcome in the Patients and Methods section and the Abstract. Yet, it is reported as a principal outcome in Table I, and is also reported in the Abstract.

The number of children of school age is given only for the placebo group (52 out of 118, Table III). But the comparisons were done for 40 in the placebo group and 42 in the antibiotic group. For 12 of 52 school age children in former, the relevant data are missing or not pertinent due to school holidays. What was the situation for the antibiotic group? Were the school age groups comparable in terms of key baseline factors? These matter are not known.

Consider now the variable therapeutic compliance. This was assessed uptil visit 3. The data in para 2 of Characteristic of Children lead us to infer that there were no missing values here and that all medicine bottles were collected. The query is: Why are there missing values for analgesic use uptil visit 3, but none for the usage of placebo/antibiotic? If the latter bottles were collected at the clinical visit 4, that fact is not consistent with the fact that 20 subjects missed this visit. Or were some bottles collected earlier and some later at visit 4? Since visit 3 admittedly occurred in a generally delayed manner for one of the groups, did that not affect the assessment of compliance as well? Why was an adjustment for this not made as was done for analgesic use?

A related vague aspect of Burke at al. concerns the persons who actually made the home visits. Were they the two authors identified as research officers, the persons thanked in the acknowledgments, the general practitioners, nurses from the general practitioners' offices, or some other persons? Was it the same category of persons for all practices, or did that vary from practice to practice, or over time? There is a distinct possibility that some of the claimed home visits did not actually occur, or occurred in a delayed and biased manner. The identification of the home visitors may hold a clue as to why that happened.

There are also concerns for the short term outcomes for which there are **no missing data**. Consider occurrence of discharging ears and treatment failure. The former is noted in Table I but not mentioned in the rest of the paper. Over what time period was it measured? If it was from the 21 day diary (aural symptoms) then the totals are not consistent with the fact that 12 diaries were not returned. There are also problems with the time period for and identification of treatment failures (see below). For contralateral pain, vomiting, diarrhea and rash, the absence of missing data is not consistent with the fact that these are parental diary derived data and only 95% of the diaries were returned.

Next consider missing data from the initial visit. We have noted that reliable data on the excluded subjects were available from only one out of the seventeen practices. Table 9 indicates the levels of missing baseline data in the two groups. Here the striking feature is that for most of these variables, we do not have the denominator that can be used to make this assessment.

At visit 2, the researcher was also to obtain "*further historical information.*" Our concerns about home visits thus apply to baseline data as well. The absent denominators possibly indicate the existence of missing values. A supportive piece of evidence is that for history of AOM, we know that 15 values were missing in the placebo group. For the 17 treatment failures in the placebo group, the number of previous episodes of AOM was not known for 4 (Table III). Also, for fever, cough and crying at baseline, the numerator and denominator are both not known; and these key variables are not directly mentioned when comparing the two groups at baseline.

These concerns further call into question the va-

lidity of the analysis for analgesic use and other compliance data, and raise the distinct possibility that missing values for some were erroneously coded as zero values (as was done for crying).

Acknowledging the problem of missing data, the authors say: "*Though data for some individual children were incomplete, the children were included in any analysis for which they were eligible. For this reason, the denominators varied.*" (Characteristics of Children, para 2).

This explanation avoids the serious problems of implementation that produced the missing data. The missing data on fever, the unequal timings for the home visits, and other points noted raise serious concerns about the completeness and quality of the data from the home visits. Unless they are clarified, the validity of ALL the researcher determined outcomes from visit 2 and visit 3 remains suspect. The data on fever at visit 2 and visit 3 and on consumption of analgesics are, in particular, not reliable or valid, and the results given for them should be discounted.

Treatment Failure

Treatment failure (TF) is an outcome for which a high statistical significance and a high odds ratio were noted. These findings are repeatedly stressed in the paper.

How was TF defined? According to the Patients and Methods, TF occurred if "*a second line antibiotic was required.*" But no time line was noted. Later in the text, we read that a TF occurred if "*a different antibiotic was started* ***on or before day 8*** *because of non-resolution or recurrence of symptoms*" (Short Term Outcome, last para). This time line includes the cases prescribed an antibiotic on the day 8 clinical examination (visit 4). But the TF data in Table I are for cases with "*[n]on-resolution or recurrence of symptoms requiring use of second line antibiotic* ***during*** *first week*" (all emphases added). In this case, children given a second line antibiotic at visit 4 would be excluded.

Including visit 4 identified cases in the count of TF is more in line with the design of the study. Yet, they seem to have been excluded from Table I. There are two more reasons to suspect this. One, at visit 4, "*15/103 (15%) in the antibiotic group and 26/110 (24%) in the placebo group showed clear evidence of clinical deterioration in one or both ears.*" (Short Term Outcome, para 3). Two antibiotic and 4 placebo cases among these had bilateral deterioration. How many of these 41 (15 + 26) were prescribed an antibiotic? If the TF counts did include them, then among the 15 such cases in the amoxycillin group, not more than two were given a second line antibiotic. This does not seem plausible.

Two, perforation of the tympanic membrane was a cause for prescribing a second line antibiotic. Perforations accounted for 2 of the 17 TFs in the placebo group. None of the 2 failures identified in the antibiotic group, on the other hand, had this as the underlying reason (Table II). Yet 16/114 (14%) of the antibiotic, and 22/118 (19%) of the placebo group had **occurrence of discharging ears** (Table I). On this issue, the Cochrane Review notes: "*it is not clear whether the "discharging ears" in Table I should be included as perforations*" [5]. Note, however, that occurrence of discharging ears was recorded over a 21 day period.

Combining the above information, then in the amoxycillin group, only at most 2 of the upto the 16 cases with possible perforations (as noted in the 21 day diary) and 15 cases with "*clear evidence of clinical deterioration in one or both ears*" by day 8 (these cases may overlap) were prescribed a second line antibiotic by day 8. A similar case can be made for the placebo group.

In an environment where general practitioners routinely gave an antibiotic to a child with AOM, this scenario is an unlikely one. For the one practice for which the data on exclusions from the study are given, we see that about half were because of an indication of antibiotic, including perforation. It is thereby more likely that treatment failures identified on the day 8 clinical examination, and who were then given an antibiotic, were not included in the TF counts, as the note in Table I states.

Another concern about TF is connected to timing and occurrence of home visits. As noted above, home visits possibly occurred at a higher rate or earlier in the placebo group; this may have led to a biased earlier identification of TF, with the antibiotic group failures being detected later and thus not counted in the revised definition. Note that 13 of the 17 or about **three quarters** of the TFs in the placebo group are vaguely classified as "*Other non-resolution*" (Table II). Did they have persistent fever? Indeed, what were the specific reasons for prescribing a second line antibiotic for these 13 cases?

The other concerns with the definition and analysis of TF are: (i) Two children (one in each group)

were withdrawn from the trial as a result of parental initiative. Were they given an antibiotic? (ii) Four children were withdrawn due to severity of cough, rash or diarrhea. Should they be deemed TF even if a second line antibiotic was not prescribed? In either case, the count of TF would be larger. (iii) In an earlier clinical trial of antibiotics for AOM, **therapeutic failure** "*was defined as remaining non-negligible symptoms (pain, fever, etc.) or insufficient resolution of infectious signs during the medical treatment period of seven days*" [7]. Under this, the TF count would possibly rise, especially in the antibiotic group, and the gap between the two groups not be as stark. (The sole recent meta-analysis for AOM using TF as an outcome has a broader definition of TF but then uses the narrowly defined data from Burke et al. [3])

For the variables **clear evidence of clinical deterioration in one or both ears,** noted by a doctor on visit 4, and **occurrence of discharging ears,** the differences between the groups could have resulted from chance variation. This is not highlighted in the Abstract or the Discussion. Yet, the narrowly defined TF is highlighted.

Suppose we deem all children identified in Table II of Burke et al. as TF. The new TF rate then is 19/118 (16.1%) in the placebo, and 6/114 (5.23%) in the antibiotic group, giving RR = 3.06, 95% CI for RR = (1.31,7.25), and chisquare p-value = 0.0078. If, on the other hand, worsened ear drum signs at visit 8 is denoted as TF (item 5 of Table I), we have RR = 1.62, 95% CI for RR = (0.91,2.89), with a chisquare p-value = 0.0933. The truth may lie between these two types of analyses.

In sum, not only is TF narrowly defined, but the time line for it seems to have been set or modified during data analysis. This problem is compounded by the lack of comparability of treatment groups at baseline, and the biased short term follow up. The analysis of TF in Burke et al. is thereby suspect, and the RR value of 8.21 is not credible. This matter is also relevant for the analytic handling of the cases with bulging ear drums at presentation. We consider this next.

Bulging Ear Drums

Now we return to children with bulging ear drums (BED) at presentation. At least 27 such cases were (mistakenly) included in the study, and were also maldistributed between the two groups. Yet the term significant difference in connection with BED does not appear anywhere. In the last paragraph of Results, the authors implicitly imply that such a difference existed, and acknowledge the need to adjust for it. We read: "*A total of 27 children with bulging ear drums were included in the study, and the data were reanalyzed after excluding these children. The results were substantially similar to those given above.*" The meaning of 'substantially similar' is not known. This method of reanalysis is also suspect, as will be detailed later.

To analyze the effect of treatment on TF (as denoted in the paper) after adjusting for BED, the following information is relevant: (i) In the antibiotic group, there were two TFs, and (ii) among the cases with TF, only one had presented with BED (last sentence, Results). Accepting these data, there are only two possible scenarios relating TF and treatment stratified by BED status at the outset. These are shown in Table 10, where x is either 0 or 1.

First, ignoring treatment, we relate TF to BED at presentation. The observed TF proportion without BED is 0.078, and with BED, it is 0.037. The RR (BED: no BED) for TF is 0.4745 with 95% CI (0.0806,2.517) and the chisquare p-value equal to 0.4421. The case for an analysis stratified by BED is not persuasive.

Nonetheless, to relate TF to treatment after adjusting for BED at outset, we tested the two possible data sets from Table 10 for homogeneity of the odds ratios with the Zelen exact test. For $x = 0$, the p-value was 1.000, and $x = 1$, it was 0.2184. We then fitted the common odds ratio (COR) model for both scenarios. In both, the exact score test p-value for the COR being unity was 0.0005, and the conditional mle of the COR equal to 8.927 with 95% mid-p CI = (2.292,58.58).

An appropriate stratified analysis adjusting for BED continues to provide evidence that amoxycillin therapy lowered the TF rate. But, and this is crucial, this analysis is predicated on a satisfactory resolution of the questions raised about the validity of the data on TF and BED, the definition of TF, and possible biases in data collection in this study.

A similar exercise can be undertaken for laterality (Table 12). We do not present it here as it does not add any new point to the discussion.

The (mistaken) inclusion of children with BED in the study appears to have led the authors to modify their description of the study population. In the Abstract, it is the children with "*mild otitis media;*"

in the Discussion, it becomes "*children ... typical ... of those with moderate symptoms and signs.*" And, instead of openly acknowledging this problem, and the failure of randomization to distribute the cases with initial BED equivalently, the authors reverse the logic of what they say in the Introduction, and argue: "*The measures of short term outcome used favored the children treated with antibiotic. This is particularly surprising in view of the fact that this group may by chance have been more severely affected – the only difference between the groups at the outset was an excess of bulging eardrums among children treated with antibiotic.*" (Discussion, para. 4).

That the first assertion is suspect has been documented already. The second one is factually flawed. Other factors in terms of which the groups were not similar at the outset were crying and possibly body weight. It is also not in line with their own earlier claim that BED is predictive of bacterial infection, making such cases more responsive to antibiotics. Under that logic, an excess of children with BED **favors the antibiotic group**. The authors here seem to be putting the best face on the multiplicity of serious problems in the implementation of the study, of missing data and in data analysis with respect to children with bulging ear drums.

Medium and Long Term Outcomes

The medium term outcomes were obtained by 1 month and 3 month tympanometry, and the long term, from a 1 year chart review. The two main outcomes at each term were consistently defined, and the follow up levels were satisfactory (Table 7). But there are some data and analysis related anomalies which need clarification.

The denominators for 1 and 3 month outcomes obtain from the numbers for the four tympanogram profiles in Figure 3. The correct placebo group total for the three month profiles is 110 and not 111, as stated at the bottom of this figure and in Table I. The total at three months in the text is 212 instead of 221; with the correction made, this becomes 220. The one and three month antibiotic group denominators in Table I are greater by a unit as they include the case with grommets, which was excluded from Figure 3. Should this case be included here, and if so, also in the numerators?

As the proportions with effusion at 1 month in the two groups were almost identical (Table I and Figure 3), the authors accord most of the text space to effusions at 3 months, which was at a borderline level of significance. Thus: "*At three months there was an excess of children with effusions in the placebo group (31 v 20), and this was accounted for largely by children with unilateral effusions (18 v eight,* $\chi^2 = 3.53$*).*"

To examine this matter, I reconstructed the data (Table 11). The chisquare test on this 2×3 table gives a p-value at 0.1002; if we combine unilateral and bilateral effusions, it becomes 0.0788. The chisquare value of 3.53 seems to have been obtained after deleting the bilateral cases, a questionable practice. But I was unable to reproduce it with the corrected or original totals, or with the continuity corrected version of the chisquare test. The authors also highlight one other difference at three months: we comment on it later.

With respect to long term outcomes, there are discrepancies between Table I and the text; what is 0.69 in the table is 0.70 in the text; and the direction for a mean difference is reversed. While such errors do not affect the inference drawn, they do indicate, at the least, an inadequate review of the manuscript drafts.

Statistical Analysis

Continuous outcomes were compared with the Mann-Whitney U test. For each such variable, the mean values by group and confidence interval for the difference were given. But group-wise standard deviations were not. So even a rough check of the computations cannot be done. The range by group is stated only for one long term outcome (mean number of recorded recurrences). The reason why it was the only one so selected is clear: here the direction of difference favored placebo therapy. By showing the ranges and other details, the point is made that the situation was not as clear cut.

For durations of pain and crying, survival curves were derived. The method used is not stated. As shown above, the survival curve based analysis for crying is **incorrect.** Figure 2 also has confidence intervals for each curve for crying at the 8 hours time point, and for pain, at 24 hours. Why these time points? What method was used to compute the intervals? What do they imply? No answers are available.

Binary outcomes were compared with the chisquare test, with an outcome measure and confidence interval given. Here some of the computations

can be checked. We find the term **odds ratio** at three places (Results part in the Abstract; last para. of Short Term Outcome; and Medium Term Outcome.) But in Table I, all the binary measures are labeled **Ratio placebo: antibiotic.** I found that all these measures, including those explicitly called odds ratios are **relative risks**, and the confidence intervals are also for relative risk. The computations are thus correct but some of the labels are not.

Whether the regular form or the continuity corrected form of the chisquare test was used is unclear. For worsening of ear drum signs at visit 4, the test results were: ($\chi^2 = 2.26, df = 2, p > 0.05$) (Short Term Outcome, para 3). This χ^2 value is for the continuity corrected form of the Pearson chisquare test. Also, the df is equal to 1, not 2. The regular chisquare p-value is 0.0933. The form of the test used elsewhere cannot be checked. At the other place where the chisquare value is stated (effusion at three months), the df is not. Here, as noted above, I was unable to reproduce the chisquare value.

The manner of reporting the analytic results is not consistent; for example, consider the p-values in Table I. For the five statistically significant values, four actual p-values are given. But for one, it is just stated that it is less than 0.05. For the twelve statistically nonsignificant values, the p-value is given for two; for nine, it is just stated to be larger than 0.05, and for one, we read it as "*NS*." Such varied reporting appears throughout the text; a confidence intervals is at times given with the p-value and at times not; sometimes only the p-value is given; sometimes a chisquare statistic is given with the df; sometimes the df is not given; sometimes counts are with the denominators and sometimes not; and so on.

For the most part, significant differences, and outcomes favoring antibiotic therapy are highlighted; the rest are downplayed. Five of the sixteen short term outcomes (fever at visit 2, treatment failure, duration of crying, analgesic use to visit 3, and days off school) were found as statistically significant difference. All five are noted in the Abstract. Of the eleven short term variables deemed nonsignificant, only the three for pain are noted, and that too in a single unclear, less informative sentence.

In addition to the main analysis with twenty variables, the authors repeated such an analysis for cases without bulging ear drums, conducted subgroup type of analyses to adjust for a variety of other factors as well as tested other hypotheses on the data from their study. The extent of the effort to find significant differences emerges from the last sentence of the Medium Term Outcome subsection: "*When the data were analyzed by laterality of onset there was no difference among children with bilateral onset or at one month but among those with unilateral onset there were effusions present in the placebo group in the ipsilateral ear at three months (26/94), 27.6% v 11/95, 11.6%; odds ratio= 2.39; 95% confidence interval 1.25 to 4.55).*" (Note the odds ratio is not an odds ratio). Given the large numbers of the outcomes and analyses done in this study, this may well be a false positive finding.

Near the end of the paper, the authors note the need to adjust for multiplicity. They defend their failure to do so by observing that all the outcomes were internally consistent and no outcome favored placebo therapy. But this is a *post hoc* observation. Moreover, for the three complications outcomes, one medium term and one long term outcome, at least the direction of effect favored placebo. Under a Bonferroni adjustment for multiplicity for just the short term outcomes, only three (failure of treatment, duration of crying, and days off school) remain significant at the 0.05 level. And as we have seen, there are serious concerns for the data quality and analysis done for all the five outcomes declared as significant in the paper.

Burke et al. also say that they used stratified analysis and intent to treat analysis as appropriate (Patients and Methods). Consider the former first. As noted, to adjust for the effect of BED, the cases with BED were deleted, and the data reanalyzed. This is not stratified analysis but a partial subgroup analysis. Above, we saw how a stratified analysis adjusting for BED can done with two possible data sets. A similar stratified analysis, based on three possible data sets, can also be done; I do not show it here, as it does not make any new point.

The effect of several factors on the probability of TF was also assessed (Table III, and Long Term Outcome, para 2). But this was done for the placebo group only; once again, a partial subgroup analysis. This study was not designed or powered to identify factors influencing the outcome in AOM. Undertaking such an analysis then is a purely fishing expedition.

For consumption of analgesic, the authors performed further analysis to adjust for, among other things, the "*interval between entry to trial and visit [3]*" (Short Term Outcome, para 2). It is not stated how this was done. Given what was done elsewhere,

it is doubtful whether an appropriate method was employed here.

Now consider intent to treat analysis. We read: "*By visit 3, 89 children (78%) in the antibiotic group and 82 (69%) in the placebo group had taken at least two thirds of the appropriate amount of their treatment.*" (Characteristic of Children, para 2). Treating cases as randomized, the "*principal analysis was on an intention to treat (pragmatic) basis.*" (Patients and Methods). Later, we read: "*A second, explanatory, analysis excluded children in the antibiotic group who had taken less than two thirds of the appropriate quantity of prescribed treatment and all patients who had received secondary antibiotic treatment.*" (Results, last but one para.)

There are three specific problem with the latter analysis. First, the exclusion of partially compliant cases for the antibiotic group only is hard to justify. Second, the removal of patients who had received secondary antibiotics is even more serious. Deleting cases based on a key outcome, and then looking at the relation between outcome and therapy may produce strange results. And three, for consumption of analgesics, an adjustment for the timings of visit 3 was done. Why was that not done for placebo/antibiotic bottles (also supposed to be collected at visit 3). I note that in adjusting for compliance, it is better to stratify by the level of compliance and examine the residual treatment effect. Other ways of adjusting for compliance also exist. [8]

Further, the authors clearly say that the denominators used in their analysis varied from outcome to outcome; and as I showed above, for some outcomes the missing levels were very high. Again this does not comport with an intent to treat analysis, which has been narrowly interpreted to only relate to compliance.

This is my basic concern: An intent to treat analysis reflects reality (not all patients fully comply with treatment regimens) and preserves the integrity of randomization. It rests on the premise of a study with equivalent groups at the outset, unbiased and thorough follow up, minimal missing data, and generally good quality. If recruitment in some centers violated the eligibility criteria, the randomization was problematic (crying and BED), follow up in the groups was not similar (visit 3 and maybe visit 2), patterns of missing data are inexplicable (fever, baseline data), and analytic methods violating the integrity of randomization are used (faulty survival curves and partial subgroup analysis), can we call the main analysis an intent to treat analysis? Does an intent to treat analysis remedy a study in which about a tenth of the cases were included in violation of one of its own criterion, and randomized with the result that the treatment groups turned out to be significantly unequal in the terms of the very factor relating to this criterion? That is my dilemma. Subgroup analysis not only produces unreliable results, reduces power and increases the type I error rate but also goes against the spirit of an intent to treat analysis. This study employed it several times, and further, used it in a partial form [9, 10].

Presentation Style

The writing and reporting style in the paper is biased towards the routine use of antibiotics for AOM in children. The bias begins from the title of the paper. In the Introduction, it is noted that prior to their study, the "*[p]ublished evidence*" on the value of antibiotics in AOM "*is conflicting.*" Nevertheless, the subtitle of the paper declares that this was a "*trial of non-antibiotic treatment in general practice.*" Thus it was not antibiotic therapy but symptomatic therapy that was on trial. Thus the scientific spirit underlying clinical trials–that an unproven active treatment be on trial–was thus turned on its head, and dominant practice was conflated with supportive evidence. If they had tested an acupuncture based treatment against placebo in China, for example, would they have written that it was the placebo that was on trial? I have yet to come across another clinical trial report with this type of a title.

This subtitle is but a marker of the biased reporting style that pervades the paper. The bias is indicated by vague presentation of baseline differences, the dismissal of the results on pain, the confused presentation of one year recurrence rate (for which the direction of effect favored the placebo), the not so clear an admission of the problems with follow up (adjustment for time to collection of analgesic bottles), the implicit admission that randomization did not produce equivalent groups (adjustment for BED, and antibiotic children more severely affected by chance), the reversal of logic for the effect of therapy on the cases with BED at presentation, the absence of explanation for high levels of missing data for fever, and so on. The manner of highlighting of significant differences and the selection of outcomes to report in the Abstract also exemplify this bias.

In the previous sections, I gave several examples of an analytic and presentation style that is not unbiased and which led me to the above conclusion. Consider another instance of bias: We read for Figure 3 that the data exclude one child with grommets. A careful comparison of this figure with Table I reveals that this case was in the antibiotic group. But this is not mentioned. Yet, in noting the relationship between TF and BED (Results, last para), an observation in relation to a single case is noted. But even this is incomplete, as it is not stated whether this case was in the antibiotic or the placebo group.

The errors of data analysis and interpretation, including the excessive search for significance, in this paper were generally such as to show better outcomes with antibiotics. As I have shown, a more careful look at the data brings all the five main supposedly significant differences into question.

Conclusion

Apart from too many outcomes and too small a sample size, the trial of Burke et al. had a generally good design. But it was beset with serious problems during the implementation phase. The problems likely began with the quality or coverage of the initial training for the study participants, and continued into the process of patient recruitment right until the stage of data analysis, interpretation and report presentation. The completeness and quality of the data at the baseline and during short term follow up is a serious concern. In the data analysis, missing values were treated as legitimate zero values, giving wrong results and conclusions. These problems were identified or inferred only by scrutinizing the various sections of the paper with care and connecting up the different statements. Even the serious implementational problems faced in the study are not clearly acknowledged, and their consequences are not addressed adequately. The style of reporting in the paper tends to gloss over them. Taking into account the totality of the information given, the numerous problems faced in the trial, and the inconsistencies I pointed out, it is difficult to justify the main conclusions drawn by of the authors.

In sum, this trial was beset with many minor and serious problems and errors. (For a summary, see Hirji (2009).) They were not just matters of interpretation of the results but related to the actual conduct of the study. In our view, the combined effect of the extent and severity of the problems detected for Burke et al. suffices to denote it a potentially **fatally flawed study**.

References

1. Burke P, Bain J, Robinson D, Dunleavey J: **Acute red ear in children: Controlled trial of non-antibiotic treatment in general practice**. *British Medical Journal* 1991, **303**:558–562.
2. Marcy M, Takata G, Chan LS, et al: **Management of Acute Otitis Media**. *AHRQ Evidence Report/Technology Assessment* 2001, **15**:?? [Agency for Healthcare Research and Quality Publication No. 01-E101, Rockville, MD].
3. Rosenfeld RM: **Clinical efficacy of medical therapy**. In *Evidence-Based Otitis Media*, 2nd edition. Edited by Rosenfeld RM, Bluestone CD, Hamilton & London: BC Decker Inc. 2003:199–226.
4. Cantekin EI: **Aggressive and ineffective therapy for otitis media**. *Otorhinolaryngol Nova* 1998, **8**:136–147.
5. Glasziou PP, Del Mar CB, Sanders SL, Hayem M: **Antibiotics for acute otitis media in children**. *The Cochrane Database of Systematic Reviews* 2004, **Art. No: CD000219.pub2**. [Doi: 10.1002/14651858.CD000219.pub1].
6. Rovers MM, Glasziou P, Appleman CL, Burke P, McCormick RA, Damoiseaux RA, Gaboury I, Little P, Hoes AW: **Antibiotics for acute otitis media: A meta-analysis with individual patient data**. *Lancet* 2006, **368**:1429–1435.
7. Thalin A, Densert O, Larsson A, Lyden E, Ripa T: **Is penicillin necessary in the treatment of acute otitis media?** In *Proceedings of the International Conference on Acute and Secretory Otitis Media*. Edited by ??, Amsterdam, The Netherlands: Kegler Publications 1986:441–446.
8. Hewitt CE, Torgerson DJ, Miles JNV: **Is there another way to take account of noncompliance in randomized controlled trials**. *Canadian Medical Association Journal* 2006, **175**:347–348.
9. Brookes ST, Whitely E, Egger M, Smith GD, Mulheran PA, Peters TJ: **Subgroup analyses in randomized trials: Risks of subgroup-specific analyses: Power and sample size for the interaction test**. *Journal of Clinical Epidemiology* 2004, **57**:229–236.
10. Montori VM, Guyatt GH: **Intention-to-treat principle**. *Canadian Medical Association Journal* 2001, **165**:1339–1341.

Tables

Table 5 - Main short term outcomes in Burke et al.

1.	Declared in Patients and Methods
	(i) Duration of symptoms, (ii) use of analgesic, (iii) clinical signs (V4), (iv) complications, (v) failure of treatment.
2.	Declared in Abstract
	(i) Diary records of pain and crying, (ii) use of analgesic, (iii) ear drum signs, (iv) failure of treatment.
3.	Reported in Abstract
	(i) Diary records of pain and crying, (ii) use of analgesic (V3), (iii) fever (V2), (iv) failure of treatment, (v) absence from school.
4.	Declared and Reported in Table I
	(i) Researcher record of pain and fever (V2 and V3) (ii) ear drum signs, (iii) discharging ear, (iv) contralateral pain (v) failure of treatment (vi) diary records of pain and crying, (vii) use of analgesic (V2 and V3), (viii) absence from school.

Note: V2 = Visit 2; V3 = Visit 3; V4 = Visit 4.

Table 6 - Baseline comparability as noted in Burke et al.

Variable	Data	Declaration	p-value∗
1. Gender	Given	A Difference	0.1523
2. Age	Not Given	No Difference	UTC†
3. Location	Not Given	No Difference	UTC
4. Social Class	Not Given	No Difference	UTC
5. Entry Season	Not Given	No Difference	UTC
6. History of OM	Not Given	No Difference	UTC
7. ENT Referrals	Not Given	No Difference	UTC
8. Prior Adenoidectomy	Numerators	Not Stated	0.2608
9. Pain Duration Before Entry	Means	Not Significant	UTC
10. Physical Signs at Entry	Not Given	No Difference	UTC
11. Bulging Ear Drums	Numerators	Not Similar	0.0189

∗1 df chisquare with full denominators; †UTC = Unable to Compute.

Table 7 - Follow up by phase of study in Burke et al.

	Numbers Missing	
Study Phase	Amoxycillin	Placebo
1. Visit 4 (Day 8)	11	8
2. Complete 21 Day Diaries	7	5
3. One Month Tympanometry	2	2
4. Three Months Tympanometry	3	8
5. One Year Record Review	4	7
Total in Group	114	118

Note: See text for explanation of items 3 & 4.

Table 8 - Missing data in Burke et al.: short term outcomes

	Number Missing			Number Missing	
Outcome Variable	Amoxycillin	Placebo	Outcome Variable	Amoxycillin	Placebo
1. Pain - V2	2	1	9. Duration of Pain	7	5
2. Fever - V2	27	25	10. Duration of Crying	8	5
3. Pain - V3	3	4	11. Analgesic Use (V2)	0	0
4. Fever - V3	63	48	12. Analgesic Use (V3)	10	7
5. Ear Drum Signs (V4)	11	8	13. Absence from School	?	10
6. Discharging Ears	0	0	14. Vomiting	0	0
7. Contralateral Pain	0	0	15. Diarrhea	0	0
8. Failure of Treatment	0	0	16. Rash	0	0
Total in Group	114	118	Total in Group	114	118

Note: Items 14, 15 & 16 deduced from the text; V2 = Visit 2; V3 = Visit 3; V4 = Visit 4.

Table 9 - Missing data in Burke et al.: baseline factors

	Number Missing			Number Missing	
Factor	Amoxycillin	Placebo	Factor	Amoxycillin	Placebo
1. Gender	0	0	9. Prior Pain	?	?
2. Age	?	0	10. Bulging Ear Drums	0?	0?
3. Location	?	?	11. Laterality	?	0
4. Social Class	?	?	12. Fever at time 0	?	?
5. Season	?	?	13. Cough at time 0	?	?
6. History of AOM	?	15	14. Crying at time 0	?	?
7. Prior ENT Referrals	?	?	15. Body Weight	?	?
8. Adenoidectomy	?	?			
Total in Group	114	118	Total in Group	114	118

Note: ? = Not determinable from the paper.

Table 10 - Bulging ear drums at outset in Burke et al.

Outcome	Placebo	Antibiotic	Total
	No BED at Outset		
No TF	$94 - x$	$93 + x$	187
TF	$16 + x$	$2 - x$	18
Total	110	95	205
	BED at Outset		
No TF	$7 + x$	$19 - x$	26
TF	$1 - x$	x	1
Total	8	19	27

Note: TF = Treatment Failure; BED = Bulging Ear Drum(s); $x = 0, 1$.

Table 11 - Effusion at three months in Burke et al.

	Effusion			
Treatment	None	Unilateral	Bilateral	Total
Placebo	79	18	13	110
Antibiotic	90	8	12	110
Total	169	26	25	220

Note: Case with grommets in antibiotic group excluded.

Table 12 - TF and initial laterality in Burke et al.

Outcome	Unilateral	Bilateral	Total
Placebo			
No TF	88	13	101
TF	14	3	17
Total	102	16	118
Antibiotic			
No TF	$96 + x$	$16 - x$	112
TF	$2 - x$	x	2
Total	98	16	114

Note: TF = Treatment Failure; $x = 0, 1, 2$.
